# Impact of Biomass Fly Ash on the Performance of Diatomite and Iron Dust Powder-Based Alkali-Activated Binder

**DOI:** 10.3390/ma19173746

**Published:** 2026-09-03

**Authors:** Darius Žurinskas, Danutė Vaičiukynienė, Karel Dvorak

**Affiliations:** 1 Faculty of Architecture and Civil Engineering, Kaunas University of Technology, Studentų st. 48, 51367 Kaunas, Lithuania; danute.vaiciukyniene@ktu.lt; 2 Faculty of Civil Engineering, Brno University of Technology, Veveří 331/95, 60200 Brno, Czech Republic; karel.dvorak@vut.cz

**Keywords:** alkali-activated binders, biomass fly ash, diatomite, iron dust, compressive strength, secondary geopolymerisation, microstructure

## Abstract

This study investigates the influence of biomass fly ash (BFA) produced from high-temperature combustion of woody biomass fuels typical of Lithuanian energy plants on the mechanical performance, microstructure, and reactivity of alkali-activated binders based on diatomite and iron dust. Diatomite was used as a reactive silica source, while iron dust served as a matrix-modifying component enhancing binder density and strength. The role of BFA (10–30%) was evaluated in terms of compressive strength, softening factor, water resistance, and structural development using XRD and FTIR analyses. The results show that the formation of a compact geopolymer gel is the key factor controlling strength development. The highest compressive strength (53 MPa) was obtained at 10% BFA; however, it decreased to 33 MPa after thermal treatment at 200 °C, indicating limited structural stability. Increasing the BFA content to 20% and 30% improved the softening factor and water resistance but significantly reduced compressive strength to below 20 MPa and 10 MPa, respectively, demonstrating a trade-off between strength and durability. XRD analysis confirmed similar mineralogical compositions in all samples, dominated by largely unreacted quartz and magnetite, while minor amounts of andradite formed after thermal treatment. FTIR results revealed increased polymerisation with higher BFA content, reflected by the shift of the Si–O–T band (~966–985 cm^−1^ to ~988–995 cm^−1^), although the presence of Ca-rich and partially unreacted phases led to a less efficient geopolymeric network. Overall, the performance of the studied systems is governed by the balance between gel formation, phase composition, and microstructural integrity, with optimal properties achieved at moderate BFA content rather than at extreme compositions.

## 1. Introduction

Recent studies have increasingly focused on the potential of biomass fly ash (BFA) as a viable precursor in alkali-activated and geopolymer binders, aiming to enhance sustainability while maintaining adequate mechanical performance [1]. Although the reactivity of untreated BFA is generally low, various modification techniques and hybrid precursor systems have been explored to improve its effectiveness in cementitious applications [1,2,3,4,5]. The following studies illustrate how different treatment methods, mix designs, and activator chemistries influence the performance of BFA-based alkali-activated materials. Hao et al. [1] modified BFA, treated by flotation to reduce carbon and increase surface area, which was used to partially replace coal fly ash in geopolymer mortar. Tests varied curing conditions (air, water, heat), activator ratios (SiO_2_/Na_2_O = 2.31 and 0.86), and BFA levels (0–60%). It was found that BFA reduced flowability but improved strength at 40% replacement under water curing with a high SiO_2_/Na_2_O ratio (63.10 MPa). Air/heat curing increased porosity and reduced strength. Higher amounts of BFA raised early drying shrinkage. In another study [2], BFA from wood was processed using calcination and ball milling to improve its reactivity. Geopolymer mortars were produced from blends of BFA from wood and coal fly ash as alumina–silicate precursors. Treated BFA from wood generally showed higher strength and lower porosity than untreated ash. High CaO content in this BFA accelerates setting and promotes early strength through geopolymerisation. XRD confirmed the formation of key crystalline phases and C–S–H gel. Lei et al. [3] demonstrated that up to 30% crop biomass ash blended with ground granulated blast furnace slag and activated by sodium silicate and carbonate produces high-strength, fast-setting, low-carbon one-part geopolymers, achieving 46.09 MPa at 28 days with an 80% lower carbon footprint than Portland cement–slag. Rajamma et al. [4] investigates the alkali activation of BFA and its blends with metakaolin using varying alkali activator compositions to optimise mechanical performance and microstructural development. Pure BFA mortars achieved 18 MPa compressive strength, while incorporating 40% metakaolin increased strength to approximately 38 MPa. The enhanced performance of blended binders is attributed to synergistic aluminosilicate reactions and the formation of additional hydrated phases. Du et al. [5] prepared a review that evaluates biomass wood ash as a precursor for alkali-activated materials. Although BWA/BFA shows low reactivity and may reduce strength at high replacement levels, combining it with more active precursors enables feasible mechanical performance. The study [6] demonstrates that incorporating up to 50 wt% BFA into metakaolin-based alkali-activated systems enhances mechanical performance by supplying additional alkaline species that promote precursor dissolution and increase the degree of geopolymerisation. However, higher BFA contents reduce the efficiency of gel formation, resulting in poorer microstructural development and diminished mechanical properties.

Biomass fly ash (BFA) generally has low reactivity because its mineral composition is dominated by quartz and calcite, which are non-reactive. However, it contains an amorphous fraction rich in CaO, which can contribute to geopolymerisation when combined with silica-rich materials like diatomite (DT) [5]. Wood ash typically contains 25–50% CaO, making calcium one of its dominant components. In our previous study [7], the chemical composition of BFA using XRF and Rietveld analysis data, showed that the amorphous part of BFA consists of CaO. Ash, enriched with reactive Ca^2+^ ions and aluminosilicate phases, acts as an effective precursor that enhances the strength development and microstructural quality of alkali-activated binders, especially when complemented by DT, which compensates for compositional limitations of BFA [7]. Feng et al. [8] demonstrated that alkaline-treated and subsequently calcined biomass power plant ash substantially enhances the strength, hydration degree, and microstructural density of alkali-activated recycled concrete. Moreover, the incorporation of GGBFS further improves mechanical performance by promoting the formation of C–S–H and C–A–S–H gels due to its high content of reactive oxides. Abdulkareem et al. [9] shows that replacing fly ash with 10–20% BFA improves early geopolymer strength, accelerates setting, and enhances microstructural development. Higher BFA contents (≥20%) reduce long-term mechanical performance and increase water absorption, while 10% replacement provides the best balance of strength, density, and durability.

DT is a porous natural pozzolanic material used in geopolymer binder systems and is rich in silica minerals, as reported by İlkentapar et al. [10]. Previous research consistently demonstrates the beneficial role of DT in fly ash-based geopolymer systems. Özsoy [11] reported that a 2% DT substitution significantly enhanced mechanical properties, abrasion resistance, and microstructural compactness, with SEM images confirming a denser matrix; although strength decreased after exposure to 300–900 °C, the highly reactive silica in DT remained suitable for geopolymer formulations. Similarly, studies incorporating larger amounts of DT (10–30%), both calcined and non-calcined, showed that reactive SiO_2_ from calcined forms improved N–A–S–H gel formation and enabled compressive strengths around 34 MPa when activated with 10–14 M NaOH [12,13]. Further findings indicate that small additions of 1–3% DT, particularly the optimal 2%, effectively fill micro-voids, refine pore structure, and enhance geopolymer strength and abrasion resistance by promoting more intensive gel development [14]. Together, these studies confirm that properly dosed DT contributes to a denser microstructure and improved mechanical performance in fly ash-based geopolymers.

The influence of iron-bearing phases and iron-derived additives on geopolymer synthesis has become an important research direction due to their potential to modify gel formation, pore structure, and mechanical behaviour [15]. Previous studies [16] on geopolymer systems have reported that the incorporation of 2–6 wt% micro- and nano-sized iron powders yields the highest compressive strengths (55–58 MPa), primarily due to the filler effect and the consequent reduction in pore volume. Recent studies provide comprehensive insights into how different iron species, their reactivity, and incorporation methods affect the performance of metakaolin- and fly ash-based geopolymer systems. Ngnintedem et al. [17] showed that substituting metakaolin with 10–20 wt.% hematite, magnetite, or goethite affected both the amorphous gel content and compressive strength of geopolymers, with 10 wt.% hematite producing the most notable improvement, despite the iron minerals remaining mostly unreacted and inducing voids and cracks that complicated strength–microstructure correlations. Complementary research on ferrisilicate zeolites demonstrated that increasing the Fe_2_O_3_/SiO_2_ molar ratio enhanced compressive strength in metakaolin-based geopolymers up to 0.8, whereas the same additions weakened clay brick-based systems, underscoring the strong dependency of Fe–silica effects on the aluminosilicate feedstock [18]. Neto et al. [19] further showed that optimised alkaline activation conditions—10 M NaOH and a Na_2_SiO_3_/NaOH ratio of 2.5—enabled effective dissolution of aluminosilicates even in low-reactivity metakaolin rich in quartz and iron, leading to structurally reliable, pumpable alkali-activated concretes. Additional studies by Ngnintedem et al. [20] found that small amounts of ferric ions obtained from dissolved hematite significantly refined pore structure and increased compressive strength to 72.80 MPa at 5 g substitution, with SEM analyses confirming reduced macro-porosity and uniform Fe dispersion within the Si–Al–Na matrix. Similarly, replacing metakaolin with 10 wt.% dissolved hematite gel or its powder markedly increased strength to 59.52 and 63.23 MPa, respectively, whereas magnetite-based additives produced slight strength reductions due to minor agglomeration and less favourable matrix densification [21]. Together, these studies highlight that iron-based additives, particularly hematite-derived reactive species, can significantly enhance geopolymer densification and mechanical performance when properly incorporated and combined with optimised alkaline activation conditions.

The aim of this study is to evaluate how biomass fly ash influences the mechanical performance, microstructure, and overall reactivity of alkali-activated binders formulated with DT and iron dust. By combining DT as a reactive silica source and iron dust as a matrix-strengthening component, the research seeks to determine whether biomass fly ash can enhance geopolymerisation processes and contribute to the development of a stronger, denser, and more efficient alkali-activated binder system.

## 2. Materials and Methods

### 2.1. Description of Source Materials

The binder precursor was prepared using three primary raw materials: biomass fly ash (BFA), diatomite (DT), and iron dust (ID). The chemical composition of each material was determined by X-ray fluorescence (XRF), and the results are presented in Table 1. The XRF analysis was performed using a Bruker X-ray S8 Tiger WD device (Bruker AXS, Karlsruhe, Germany), which uses a rhodium (Rh) tube at a voltage of up to 60 V and a current of up to 130 mA. These materials were selected due to their high oxide content and potential to contribute to the formation of reactive aluminosilicate and ferric phases within the binder system.

Biomass fly ash from a local Lithuanian biofuel boiler plant exhibited a heterogeneous chemical profile typical of ash derived from biomass combustion. The dominant oxides were SiO_2_ (32.99%) and CaO (32.63%), which together constituted the majority of the material. Moderate quantities of K_2_O (7.48%), Al_2_O_3_ (5.91%), MgO (5.57%), and SO_3_ (5.38%) were also detected. This combination of siliceous and calcareous components suggests that BFA may act both as a reactive precursor and as a source of alkaline species that can influence the binder’s activation process [22]. In other scientific studies, BFA has also been used as a component of precursor blends.

DT was obtained from a Lithuanian manufacturer and subjected to calcination at 800 °C to enhance its reactivity [23]. Following thermal treatment, the material was ground and sieved through a 0.63 mm mesh to ensure a uniform particle size distribution suitable for structural and reactivity analyses. XRF analysis confirmed that calcined DT was predominantly composed of SiO_2_ (85.05%), reflecting the highly siliceous nature of diatomaceous earth. Minor amounts of Al_2_O_3_ (7.74%) and Fe_2_O_3_ (3.18%) were present, while all remaining oxides were below 2%. The high silica content indicates that DT serves primarily as a reactive siliceous component within the binder formulation.

Iron dust is an industrial by-product generated in Lithuanian shipbuilding facilities, where various metalworking, surface preparation, and finishing operations—such as cutting, grinding, sandblasting, and corrosion-removal processes—produce fine particulate residues enriched in iron oxides and metallic Fe. This by-product stream is typically characterised by high iron content and a heterogeneous particle-size distribution, making it a potentially valuable secondary raw material for use in alkali-activated binders, geopolymers, or other sustainable construction applications, provided that its chemical composition, mineralogical phases, and environmental safety are thoroughly assessed. Iron dust consisted almost entirely of Fe_2_O_3_ (97.10%), with all other oxides collectively accounting for less than 2%. This exceptionally high iron oxide content identifies ID as a nearly pure ferric source, which may influence the binder’s structural development, densification, and potential interactions during hydration.

To evaluate the suitability of raw materials for alkali-activated binder production, it is important to know not only their chemical composition but also their mineralogical composition, which was determined according to X-ray diffraction analysis. For XRD analysis, A Bruker D8 Advance X-ray (Bruker AXS, Karlsruhe, Germany) diffractometer was used, which performed scans using Bragg–Brentano geometry with a scanning step of 0.02 degrees. The X-ray beam was generated by CuKα with a Ni filter, operating at up to 60 V.

**Figure 1:**
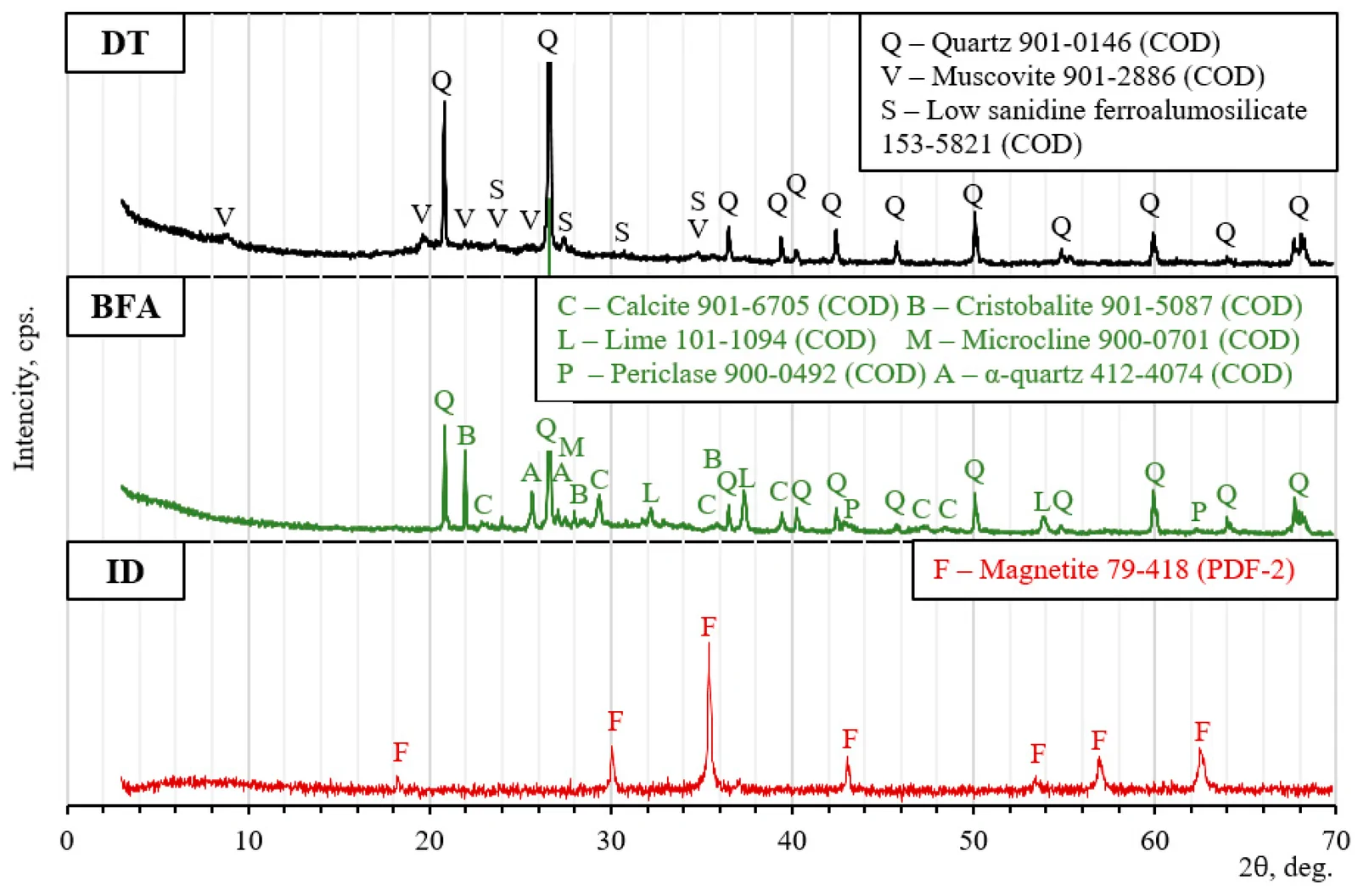
Mineral composition of the raw materials, determined by XRD analysis. DT—diatomite, biomass fly ash (BFA), and iron dust (ID).

The crystalline composition of DT was primarily dominated by quartz and muscovite, while sanidine appeared only as a minor phase (Figure 1). Quartz and muscovite are typical constituents of natural DT, reflecting its sedimentary origin. In contrast, the occurrence of sanidine—a high-temperature potassium feldspar—indicates mineralogical transformations induced by thermal treatment. Sanidine forms during the calcination of DT at around 800 °C, when the original aluminosilicate components partially melt and recrystallise into feldspathic phases [24]. The presence of quartz, muscovite, and sanidine in the calcined DT sample is consistent with results reported by Figarska-Warchoł et al. [25], who observed a similar mineralogical assemblage in thermally treated DT, confirming that high-temperature processing leads to the formation of comparable feldspar-type phases alongside preserved primary minerals.

Mineralogical XRD analysis revealed that magnetite was the sole crystalline phase present in the iron dust (ID), indicating that this well-defined iron oxide mineral represents the only detectable crystalline component (Figure 1).

The mineral composition of biomass fly ash (BFA) is primarily dominated by quartz, which constitutes the main crystalline phase identified in the material. A portion of the calcium oxide present in the ash has undergone carbonation, resulting in the formation of calcium carbonate. Additional crystalline phases, such as microcline and periclase, were also detected. A similar mineralogical profile for biomass-derived ashes has been reported by other researchers [26], where quartz and calcium-bearing compounds were likewise identified as the predominant crystalline constituents.

**Figure 2:**
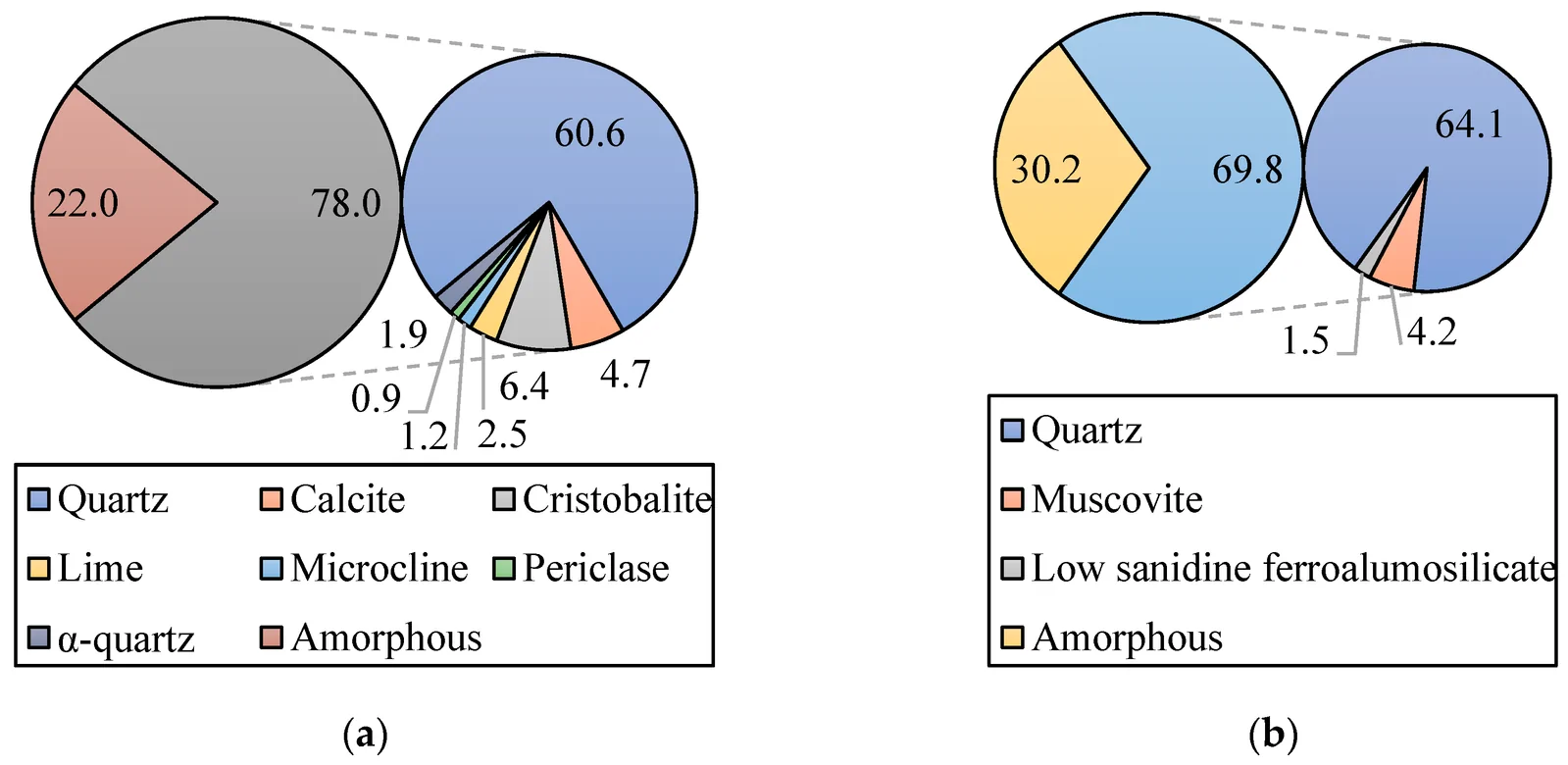
Mineral composition determined by Rietveld analysis (wt%): (**a**) BFA and (**b**) DT. The grey dashed line represents the division of the total crystalline phase into the quantities of individual crystals.

The results of the quantitative analysis of BFA and DT in the starting materials obtained by XRD, using the Rietveld analysis method, are presented in Figure 2. A comparison of the chemical compositions of the XRD minerals obtained by Rietveld analysis with the XRF results (Table 1) revealed that the amorphous phase of BFA consists of lime, while DT consists of SiO_2_.

**Figure 3:**
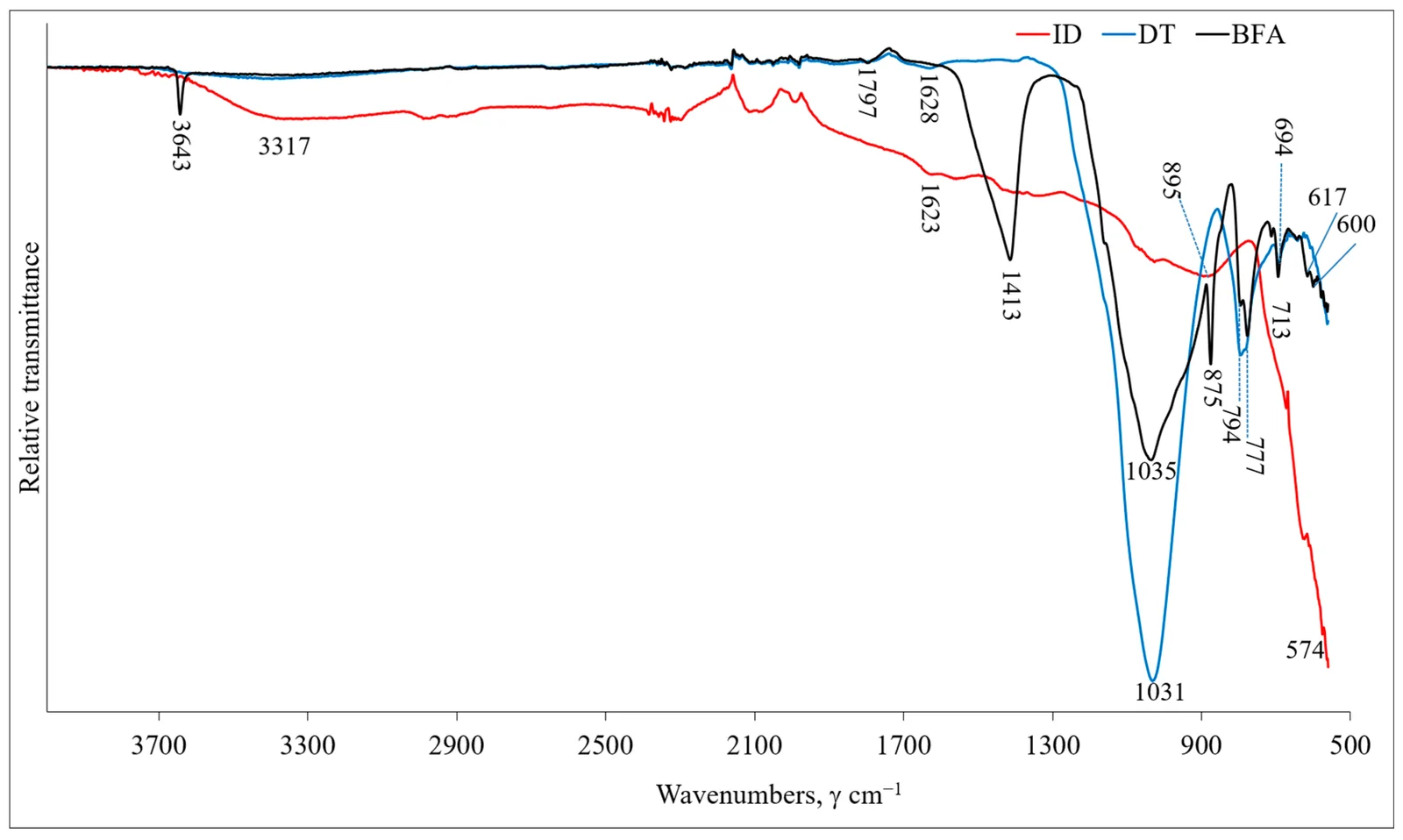
FT-IR curves of initial materials for alkali-activated binder precursor.

FTIR spectroscopy was used alongside XRF and XRD analyses to better clarify the chemical and structural properties of the initial precursor materials: biomass fly ash (BFA), calcined DT, and iron dust (ID). IR spectroscopy was performed using the Perkin Elmer FTIR Spectrum GX spectrophotometer. The spectra were recorded in the wavenumber range 4000–650 cm^−1^, with an average of 64 scans at a wavenumber resolution of 1 cm^−1^ at room temperature. The FT-IR spectra clearly reflect the distinct origins, compositions, and functional roles of these materials in alkali-activated binder systems (Figure 3). Calcined DT is characterised by a strong dominance of silica-related vibrational modes; biomass fly ash exhibits a mixed spectral signature associated with both siliceous and calcareous phases, while iron dust displays absorption bands typical exclusively of iron oxides.

The FT-IR spectrum of biomass fly ash reveals several characteristic absorption bands that are consistent with its heterogeneous chemical and mineralogical composition. A sharp band observed at approximately 3643 cm^−1^ is attributed to O–H stretching vibrations associated with portlandite (Ca(OH)_2_), indicating the presence of calcium hydroxide in the ash. The intense band centred around 1035 cm^−1^ corresponds to asymmetric Si–O–Si stretching vibrations, which are characteristic of quartz. This assignment is further supported by the presence of bands at approximately 794 and 777 cm^−1^, corresponding to symmetric Si–O stretching vibrations typical of crystalline silica. In addition, bands observed near 875 cm^−1^ and 1413 cm^−1^ are attributed to CO_3_^2−^ bending and asymmetric stretching vibrations, respectively, confirming the presence of calcium carbonate formed through the carbonation of free CaO. These FT-IR features confirm that BFA contains a combination of siliceous and calcareous phases, in agreement with the XRD results that identified quartz, Ca(OH)_2_, CaCO_3_. The simultaneous presence of silicate and carbonate bands indicates that BFA can function as a partially reactive precursor.

In contrast, the FT-IR spectrum of calcined DT is dominated by vibrations related to silica. A very intense band at approximately 1031 cm^−1^ corresponds to asymmetric Si–O–Si stretching vibrations, which are characteristic of both amorphous and crystalline forms of silica and reflect the highly siliceous nature of the material. A band located around 794 cm^−1^ is attributed to symmetric Si–O stretching vibrations associated with quartz. Additionally, the band observed near 1628 cm^−1^ is related to O–H vibrations and H–O–H bending modes, indicating the presence of physically adsorbed water within the porous structure of the calcined DT. These spectral features confirm that DT is predominantly composed of silica and possesses a high potential as a reactive siliceous component in alkali-activated systems.

The FT-IR spectrum of iron dust exhibits a markedly simpler character compared to BFA and DT, reflecting its nearly pure iron oxide composition. A strong absorption band observed in the range of approximately 574 cm^−1^ is attributed to Fe–O stretching vibrations characteristic of magnetite. This observation is fully consistent with the XRD results, which identified magnetite as the sole crystalline phase present in the iron dust. In the higher wavenumber regions (around 3400 and 1630 cm^−1^), weak and broad signals are observed, which can be associated with O–H stretching and bending vibrations of surface-adsorbed water.

The granulometric composition of the precursor is significant for the formation of hydration products after alkali activation.

**Figure 4:**
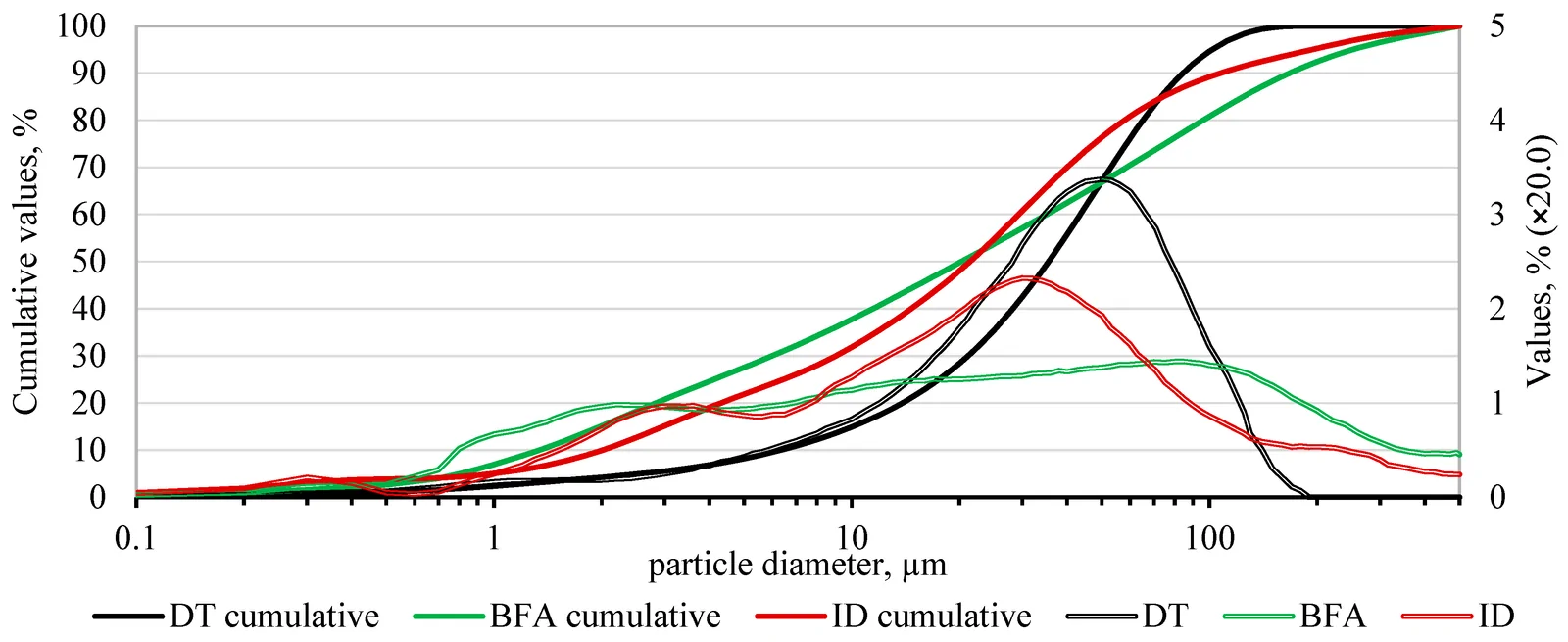
Particle size distribution of the raw materials DT, BFA, and ID.

The results of the particle size analysis are summarised in Table 2. To determine the particle size distribution, a ‘Cilas 1090’ laser particle size analyser (manufactured by ‘Cilas laserandbeyond’, Orléans, France) was used. The device can perform measurements in the range of 0.1–500 μm using dry dispersion mode. The BFA fraction exhibited the broadest particle size distribution, ranging from approximately 0.04 µm to over 400 µm, without distinct peaks in the particle size distribution, indicating a wide and heterogeneous range of particle sizes (Figure 4). Similarly, the ID fraction also showed a broad particle size distribution extending from about 0.04 µm to more than 400 µm; however, in contrast to BFA, the ID distribution displayed a bimodal character, with pronounced peaks centred around approximately 12–15 µm and 45–50 µm, reflecting the presence of both fine and coarser particle populations. In comparison, the DT fraction exhibited a noticeably narrower particle size distribution, spanning from approximately 0.04 µm to about 130 µm, with a distinct and sharp peak near 45 µm, indicating a more uniform particle size composition.

The finer particle size of ID, reflected by its lower d_10_ and d_50_ values relative to DT, is consistent with its higher specific surface area (3754 cm^2^/g), which is comparable to that of BFA and significantly exceeds that of DT (Table 2).

**Figure 5:**
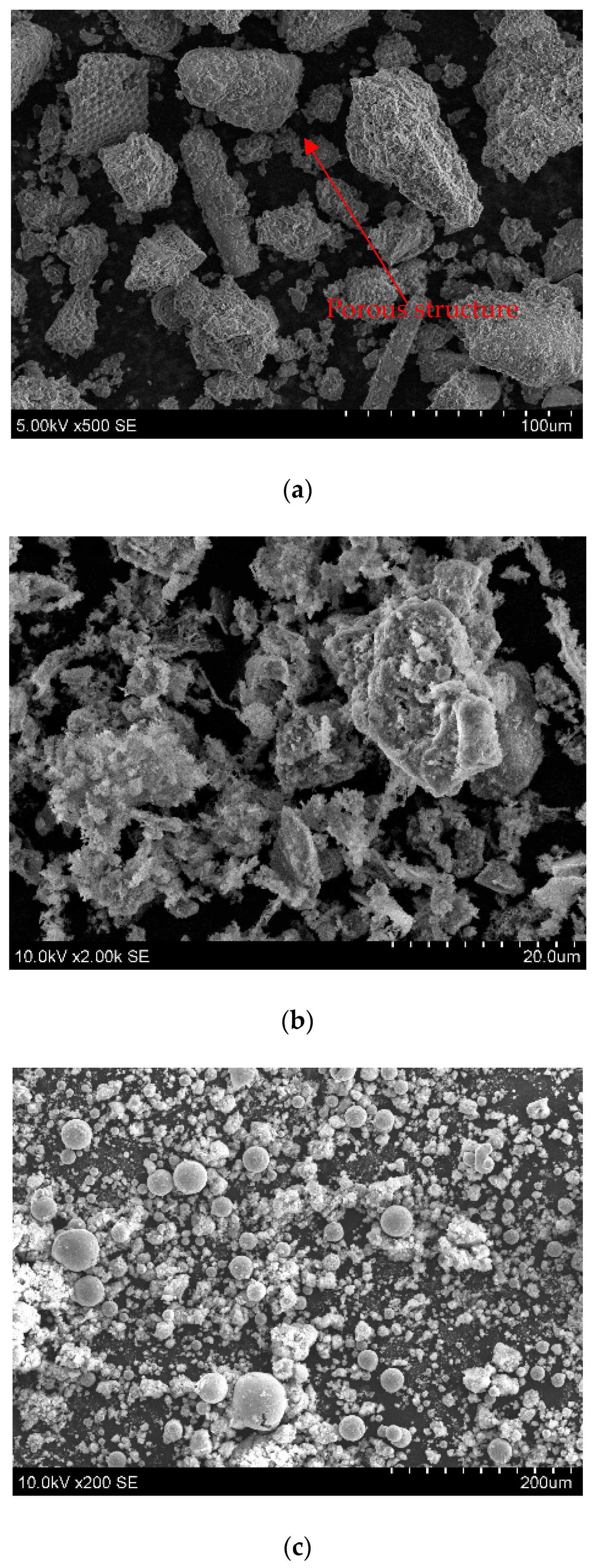
Microstructure (SEM images) of DT (**a**) and BFA (**b**) and ID (**c**).

Figure 5 shows the scanning electron microscopy (SEM) microstructures of the DT, BFA and ID powder fractions. The microstructure was determined using a scanning electron microscope (SEM); Hitachi S-3400 N Type II (Hitachi, Tokyo, Japan). The microscope provides high-resolution images with acceleration voltages of 5 V and 15 V. Based on SEM images, clear morphological differences are observed among the samples, reflecting their distinct formation and processing histories. The DT powder (Figure 5a) consists of relatively larger particles than BFA, as confirmed by the particle size analysis, and shows predominantly rounded shapes with comparatively porous surfaces. The reduced angularity suggests a different formation mechanism relative to BFA. The smoother morphology corresponds to the lower specific surface area measured for this fraction and may result in more uniform packing. A morphology comparable to that observed in the DT fraction, characteristic of fossil-algae–derived DT, has been reported by Zuluaga-Astudillo et al. [27]. The BFA powder (Figure 5b) contains highly irregular and angular particles with sharp edges and pronounced surface roughness. Such morphology is typical of mechanically comminuted materials and is consistent with the broad particle size distribution and high specific surface area measured for this fraction. The rough faceted surfaces may enhance reactivity and influence packing behaviour during consolidation. The ID powder (Figure 5c) displays a transitional morphology, containing both angular and partially rounded particles. The surface texture is moderately rough, and the particle size distribution lies between that of BFA and DT. This intermediate microstructural character aligns with the measured granulometric parameters and suggests that ID may exhibit hybrid behaviour in terms of flow, packing and sintering response.

### 2.2. Sample Preparation

Table 3 presents the mix designs used for producing alkali-activated materials based on biomass fly ash (BFA), iron dust (ID) and diatomite (DT). Three series of mixtures were prepared by varying the BFA:DT mass ratios (1:9, 2:8 and 3:7), while the amount of ID was adjusted accordingly to maintain a total precursor content of 100 wt%. Within each series, nine mixtures were formulated by systematically changing the alkali activator dosage and concentration. The alkali activator consisted of a laboratory-prepared sodium hydroxide solution, made by dissolving NaOH pellets in water to the required concentration. In addition, the compositions of the samples were calculated to ensure that the resulting molar ratios were Si/(Al + Fe) = 1.5, 2, and 2.5; (Na + Ca)/(Al + Fe) = 0.7, 1, and 1.3. These molar ratios were selected because they define the optimal balance between silicate species, charge-balancing cations, and the aluminosilicate framework, which directly governs geopolymerization efficiency [7,28].

This systematic variation in precursor proportions and activator parameters enabled a comprehensive assessment of how BFA content, DT reactivity and ID addition influence the alkali-activation behaviour and the resulting mechanical properties.

**Figure 6:**
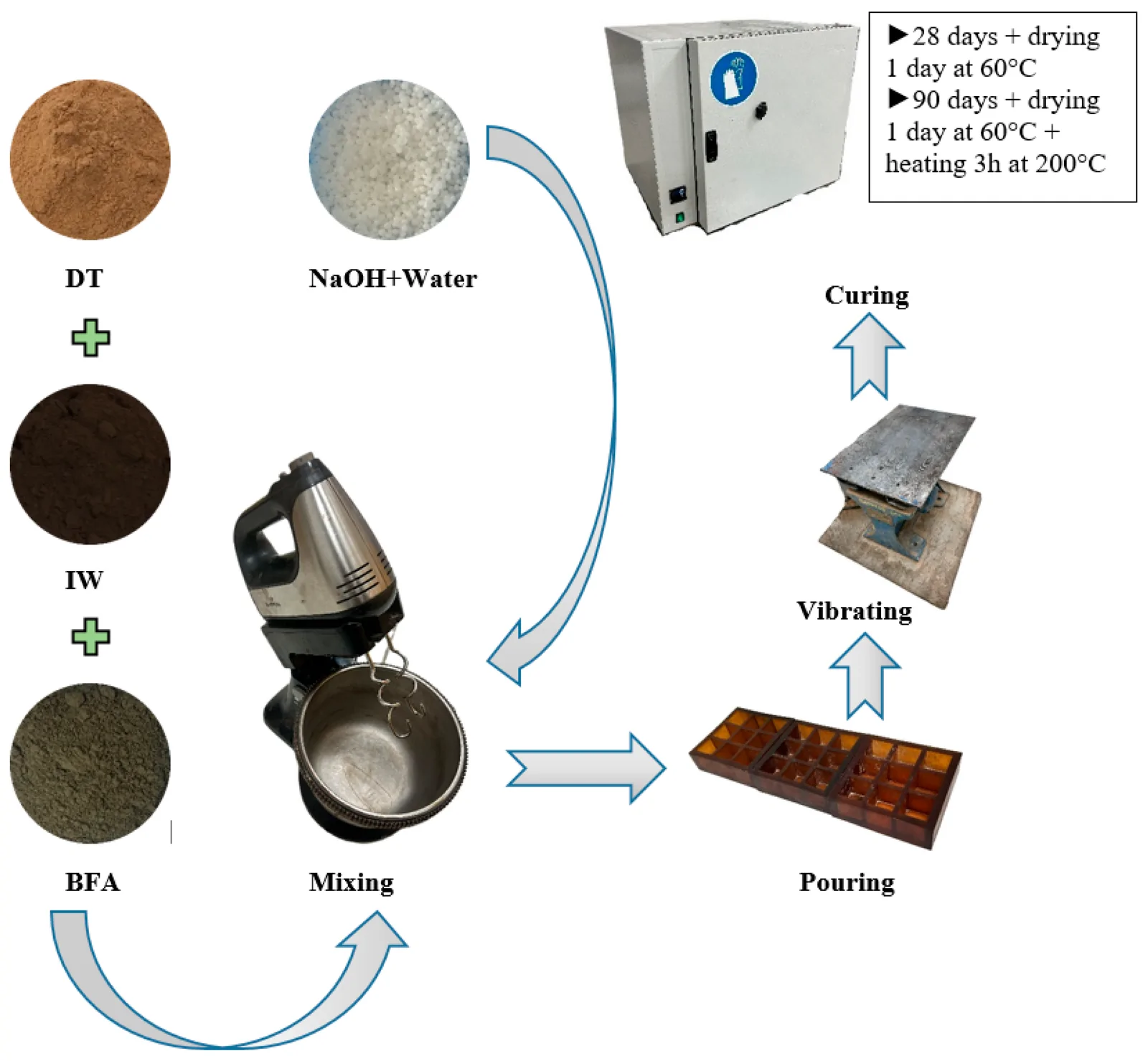
Order of the sample preparation steps. The arrow 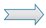 indicates the order in the preparation of the samples. The arrow ► refers to the curing conditions for the test samples.

The samples were prepared by first dry-mixing the DT, BFA and ID powders to ensure uniform distribution of the solid precursors according to the scheme shown in Figure 6. The required amount of alkali solution was then added gradually, and the mixture was blended using a hand-held rotary mixer until a homogeneous and workable alkali-activated paste was obtained. The fresh paste was cast into 20 mm × 20 mm × 20 mm cubic molds and compacted on a vibrating table to eliminate entrapped air. After casting, the molds were sealed in plastic bags to prevent moisture loss during the initial curing stage.

Two curing regimes were employed. In the first regime, the samples were kept under ambient conditions for 24 h and subsequently pre-cured at 60 °C for at least 24 h. After this initial curing period, the samples were demoulded and stored for 28 days prior to testing. In the second regime, the hardened 90-day samples were additionally subjected to thermal treatment, being heated to 200 °C for a minimum of 3 h to assess the influence of post-curing heat exposure on material performance.

Before softening and compressive strength measurements, the samples were dried at 60 °C for a minimum of 24 h.

### 2.3. Experimental Methods

The XRD analysis of the samples was performed using a Panalytical Empyrean diffractometer (Malvern Panalytical B.V., Almelo, The Netherlands). The Θ–Θ reflection Bragg–Brentano para-focusing geometry was used. The device was equipped with a Cu anode (λ = 1.54184 Å), programmable divergence slits and a PIXcel3D detector with 255 active channels. The line scanning mode was used with X-ray generator settings of 45 kV and 40 mA. The measured range was 5–70° with a step size of 0.013° and 157 s per step. The total measurement time for each sample was 50 min.

The mineral composition was analysed using software: Oxford Cryosystems Crystallographica Search-Match (version 2), which uses the ICDD PDF-2 database, and Institute of Crystallography-CNR-Bari QUALX2.0 with the COD database.

Compression tests are performed using a computerised press, model “ToniTechnik 2020.0600/132/02 (Toni Technik Baustoffprüfsysteme GmbH, Berlin, Germany)”. Average compression strength values are obtained from the average of at least 3 samples.

The softening coefficient is used to evaluate the durability, water resistance, and structural integrity of alkali-activated materials. This parameter expresses how much the compressive strength of a sample decreases after water saturation compared to its dry state. It is calculated according to Equation (1):

(1) $$K=\frac{C_{w}}{C_{d}}$$

Here, $C_{w}$ denotes the compressive strength (MPa) of samples cured for 28 days and subsequently immersed in water for 24 h, while $C_{d}$ represents the compressive strength (MPa) of samples cured for the same period and then dried for 24 h.

Samples for detailed chemical (XRD, FT-IR) and microstructural (SEM) analysis were selected based on the highest softening factor (water resistance) and the highest compressive strength. For the FT-IR and SEM analyses, the equipment and configuration described in the section on the initial materials were used.

## 3. Results and Discussion

### 3.1. Mechanical Properties and Softening Factor of Alkali-Activated Binders with BFA

The mechanical properties of the samples are investigated in relation to compressive strength and resistance to water exposure. Compressive strength is determined after 28 and 90 days for three groups, which are separated according to the BFA content in the samples. The compressive strength test was carried out in accordance with the European standard [29], using a loading rate of 0.6 MPa/s for a reference area of 4 cm^2^.

**Figure 7:**
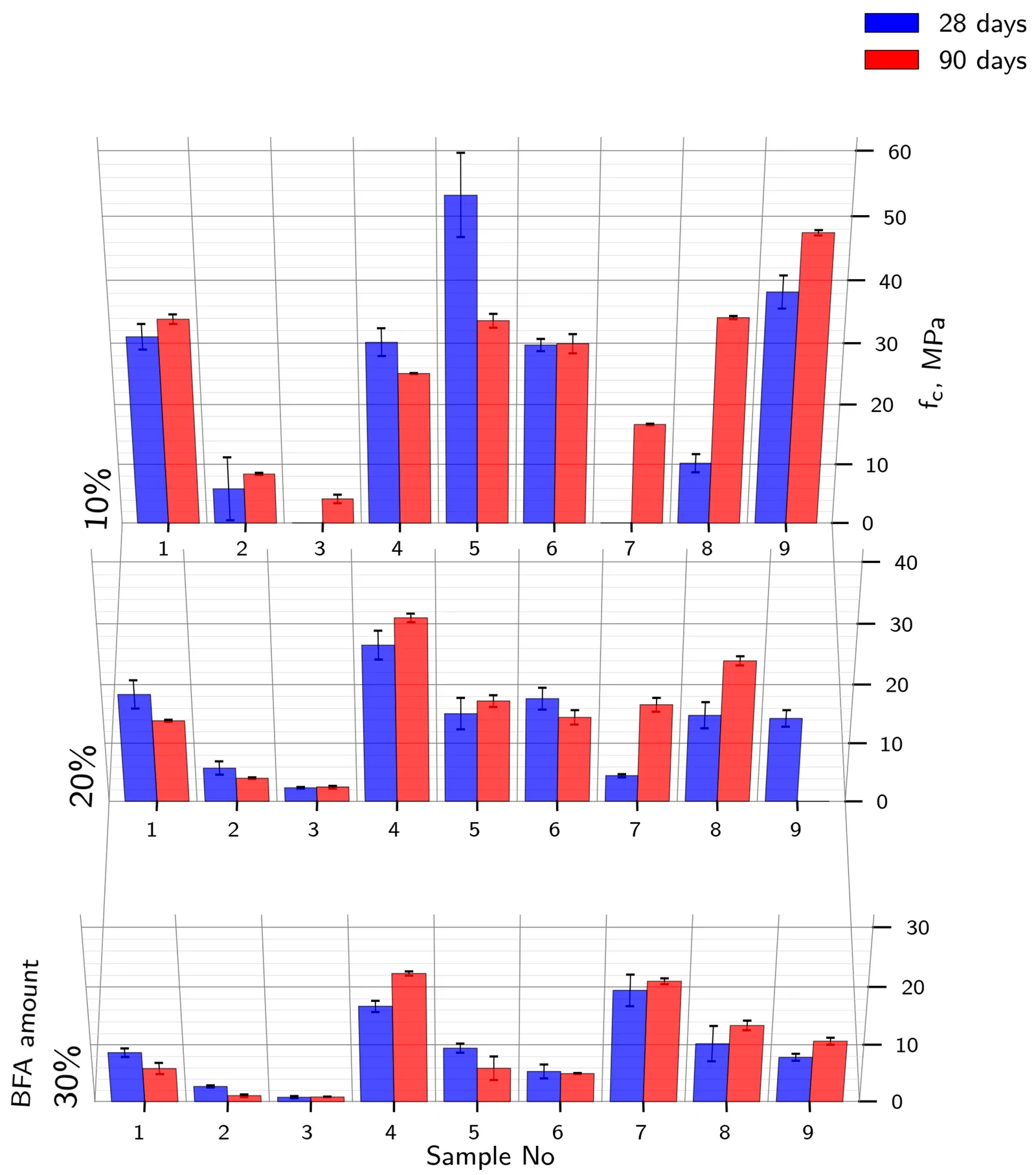
Results of the compressive strength of the samples after 28 and 90 days, with BFA content of 10%, 20%, and 30%.

In the compressive strength results (Figure 7) for cured samples aged for 28 days and 90 days, a significant decline in strength was observed only in sample 10-5, where the average compressive strength decreased from 53 MPa to 33 MPa. Explaining the cause of this phenomenon is, in principle, quite complex, as it could have been caused by several processes. In contrast to low-calcium alkali-activated systems, which typically exhibit slow curing and require additional heat [30,31], the binders studied here contain a Ca-rich biomass fly ash (32.6 wt% CaO). Therefore, the reduced strength and limited structural stability observed at higher BFA contents are more likely related to an insufficient NaOH/(BFA + ID + DT) ratio and the presence of partially unreacted Ca-bearing phases, rather than to a lack of calcium. This interpretation is supported by XRD/FTIR evidence of residual quartz and carbonate phases. All samples were cured under identical conditions; therefore, heating timing did not affect strength. The 90-day thermal treatment was chosen to assess long-term stability rather than accelerate curing. Because the materials react slowly, late heating demonstrates how the matured gel network withstands thermal exposure, reflecting durability rather than curing kinetics. When Fe is present in the system, these processes proceed more slowly [32,33]. Moreover, the heating regime prior to compression is also decisive, as thermal expansion and contraction (loss of free water) may cause cracking [34].

It is important to focus on the results obtained for samples 10-8 and 10-9 after 28 and 90 days of curing, respectively, based on their alkalinity. The compositions of samples 10-5 and 10-8, and 10-6 and 10-9, are identical, but the change in compressive strength after 28 and 90 days is significantly different. In the first case (10-5 and 10-6), where the alkalinity of the composition was lower, the compressive strength decreased or remained unchanged, even though the samples were heated after 90 days; however, for samples (10-8 and 10-9), which contained more alkali, the compressive strength increased significantly—this can be explained by the fact that a higher alkali content leads to the formation of more gel [35].

The following analysis examines the effect of BFA on compressive strength and the interaction between CaO and NaOH. Regardless of the individual samples, the fact that compressive strength decreases with increasing CaO content (BFA content) can be associated with the expanding effect of CaO, increased porosity, and the formation of weaker Ca compounds [36]. However, when considering the samples with the highest average compressive strength from the different BFA groups, it can be observed that in the sample series 4-5-6 and 7-8-9, as the BFA content increases, the highest compressive strength shifts toward higher alkalinity, but the NaOH ratio relative to the source materials remains practically the same. Upon analysing the composition of these series in different BFA groups, it can be observed that in series 4-5-6, the highest compressive strength corresponds to an NaOH ratio to the source materials of 0.11–0.12, while in series 7-8-9, the NaOH ratio is 0.14–0.15. It can be concluded that an increase in the amount of BFA significantly reduces the strength. The Ca cations in BFA do not substitute for Na cations, as FTIR analyses indicate that calcium mainly forms carbonate and hydroxide phases (CaCO_3_, Ca(OH)_2_) rather than participating in alkali activation. Consequently, Na^+^ remains the primary charge-balancing ion in the geopolymer network, while Ca^2+^ contributes to secondary C–S–H or C–A–S–H formation, consistent with previous studies [7,9,36]. This explains why the maximum strength depends strongly on the activator-to-mixture ratio rather than on calcium content.

**Figure 8:**
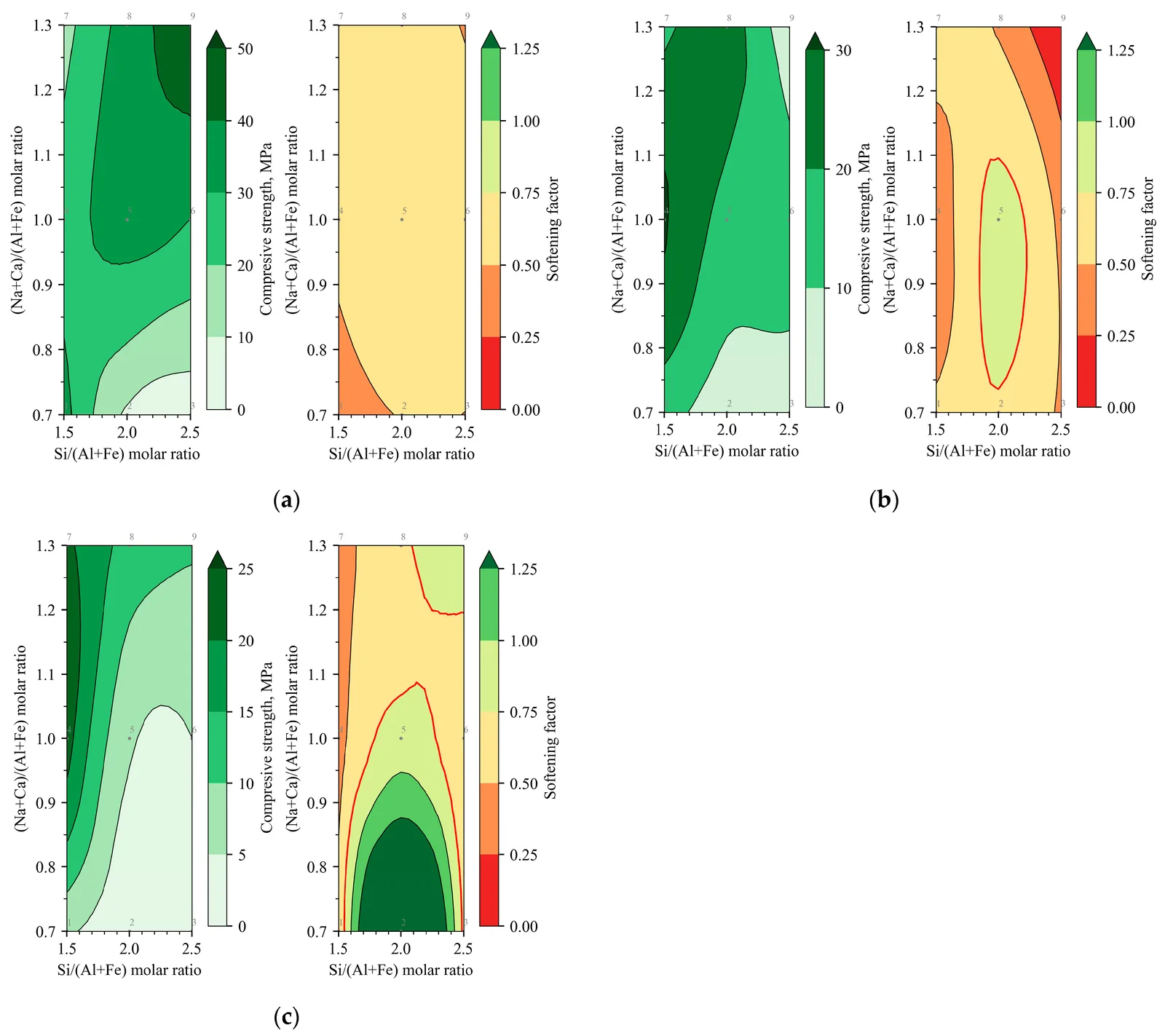
The dependence of the compressive strength and water resistance factor of the samples after 90 days on the molar ratios when the BFA content is (**a**) 10%, (**b**) 20%, and (**c**) 30%. The small grey numbers refer to the sample number.

However, based on the diagrams in Figure 8, which show the dependence of compressive strength and the softening (water resistance) factor on the molar ratio, several observations can be made regarding the samples that were cured for 90 days and heated at a temperature of 200 degrees for at least 3 h. The softening factor was determined as the ratio of compressive strength after 24 h water immersion to that of oven-dried samples, in accordance with the European standard [37]. Results for the water softening factor are not provided for samples after 28 days of curing—they did not hold their shape after the test. The vertical axis of the diagrams shows the molar ratios of Ca and Na relative to the sum of the moles of Al and Fe. This means that as the BFA content increases, the number of Ca moles increases and, at the same time, the number of Na moles decreases, although the ratio remains the same. However, in this case, the amounts of Fe and amorphous Si in the DT decrease slightly. The horizontal axis shows the ratio of Si moles to the sum of Al and Fe moles—which means that as the BFA content increases, the amorphous Si in the DT is replaced by BFA’s mineral Si, which is relatively inert.

Analysing the dependence of compressive strength on the molar ratio, the regions of maximum compressive strength vary, as mentioned above, depending on the molar content of amorphous Si and Na. At a BFA content of 10%, the highest compressive strength is observed in the zone where the Si/(Al + Fe) ratio is 2.3–2.5 and the (Na + Ca)/(Al + Fe) ratio is 1.2–1.3. By increasing the BFA content to 20%, the ratios of Si/(Al + Fe) and (Na + Ca)/(Al + Fe) at which the maximum compressive strength occurs decreased to 1.5–1.7 and 0.95–1.05, respectively, due to the reduced amounts of reactive Si and Na. Upon further increasing the BFA content by 10% to 30% of the DT content, the maximum compressive strength shifted more sharply toward the left boundary. This would mean that the maximum compressive strength was not achieved based on the Si/(Al + Fe) ratio and lies even further to the left, or that it was achieved, but the optimal (Na + Ca)/(Al + Fe) ratio remained practically unchanged from a 20% BFA content—this could indicate that the C-S-H forms more rapidly than the N-A-S-H gel [36]. In any case, this is sufficient to create a general pattern of change in compressive strength based on molar ratios: compressive strength depends on the amounts of amorphous Si and Na.

Based on the obtained values of the softening factor and molar ratios, the most stable samples are those with Si/(Al + Fe) and (Na + Ca)/(Al + Fe) ratios of approximately 2 and 1, respectively; these ratios match the optimal ratios obtained for geopolymers [38]. Although at a BFA content of 30%, the optimal softening factor for the (Na + Ca)/(Al + Fe) ratio is lower, this can be disregarded in this case because the baseline compressive strength values are very low and the errors can be very large. In general, considering the compressive strength and stability from a practical standpoint, at 10% BFA, the compressive strength is sufficient, but the resulting binder is not stable. Adding more BFA (20% and 30%), although the binder is stable, the average compressive strength is quite low (less than 20 and 10 MPa).

### 3.2. XRD and FT-IR-Based Mineral Composition of Alkali-Activated Binders with Different BMA Contents

**Figure 9:**
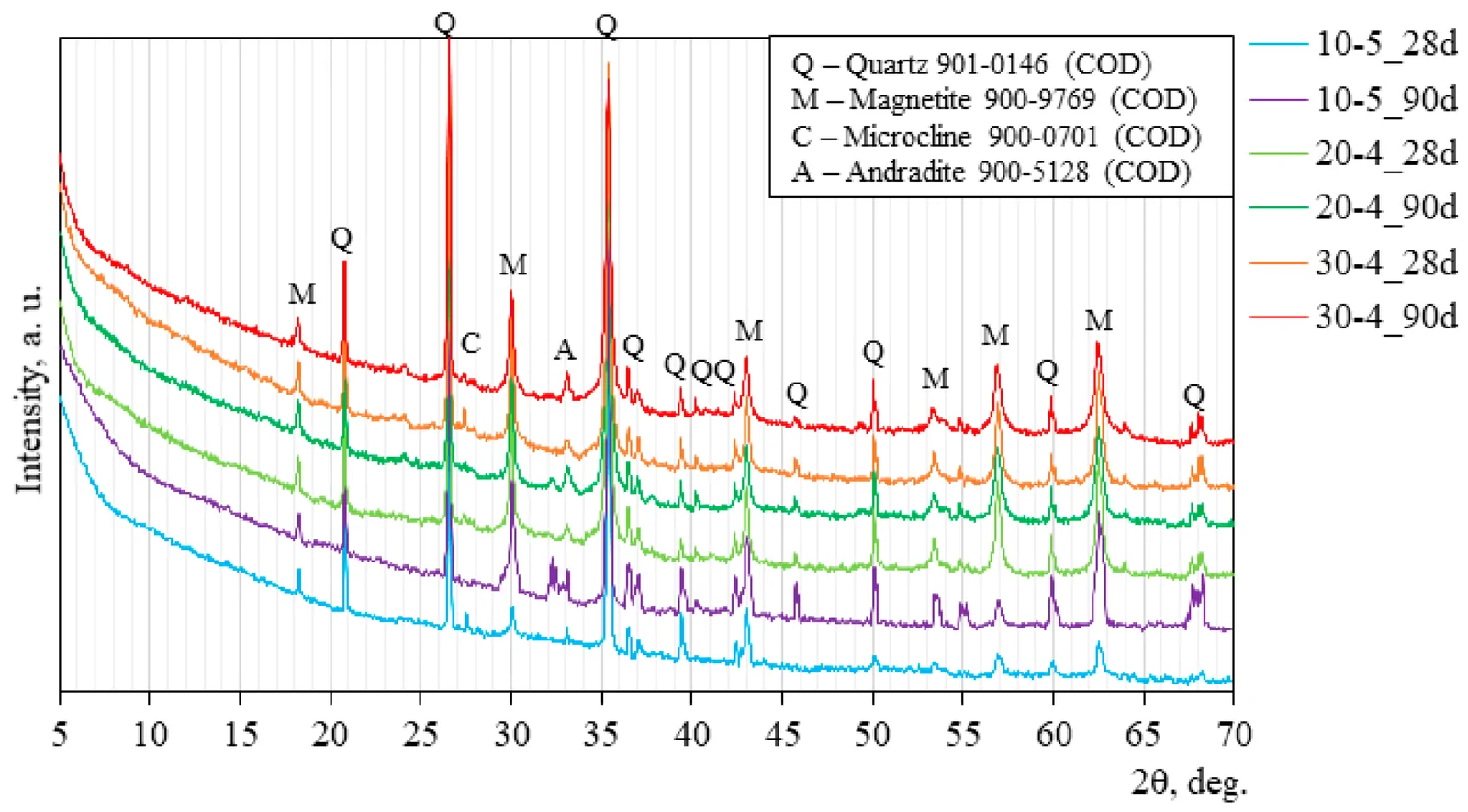
XRD patterns of alkali-activated binders (10-5, 20-4, 30-4) with different amounts of BFA content.

Three sample types (10-5, 20-4, 30-4) exhibiting the highest strength values within each series containing different proportions of BFA ash were selected, and their mineralogical composition was subsequently determined (Figure 9).

XRD analysis showed that all samples presented almost identical diffraction patterns, indicating similar mineralogical compositions. Quartz and magnetite were identified as the dominant crystalline phases and remained unreacted from the precursors (ID, BFA, and DT) following alkaline activation. The absence of significant changes in the XRD patterns after thermal treatment at 200 °C suggests that such elevated temperatures did not significantly affect the crystalline phase assemblage of the materials. Yousefi et al. [39] found that quartz remains in the matrix unreacted, as mechanically rigid particles, retaining its crystalline structure and acting primarily as a passive filler rather than a reactive phase. Wu et al. [15] demonstrated that magnetite in a geopolymer matrix generally acts as a relatively stable and weakly reactive filler phase. Its participation in chemical reactions is limited; nevertheless, magnetite may contribute to microstructural densification and an indirect increase in mechanical strength. However, owing to its mixed Fe^2+^/Fe^3+^ oxidation states, magnetite may not behave solely as a passive filler but can also act as an active participant in the reaction processes, thereby influencing the geopolymerisation pathway.

A minor amount of the newly formed crystalline phase—andradite—was identified in this study. The intensity of peaks attributed to andradite was higher in thermally treated samples (10-5_90d; 20-4_90d; 30-4_90d) than in untreated samples (10-5_28d; 20-4_28d; 30-4_28d), indicating that elevated temperature enhances the degree of ID reactivity during alkaline activation. The formation of andradite reflects ongoing mineralogical transformations and may positively influence mechanical properties by acting as an additional stiffening component within the matrix. Consequently, the overall strength development observed in this study can be attributed to a complex interplay of factors, including the extent of gel formation, porosity evolution, thermally induced microstructural changes, and the formation of new crystalline phases.

Similar findings were reported by Xu et al. [40], who demonstrated that alkaline activation induces complex chemical and mineralogical transformations in copper slag. Andradite, identified as a calcium–iron silicate garnet (Ca_3_Fe_2_(SiO_4_)_3_), forms during hardening and curing under alkaline conditions. Its formation is attributed to the high Fe and Ca content of copper slag, combined with sufficient Si availability in the activated system. The formation of the Fe-bearing andradite crystalline phase further contributes to the evolution of mechanical properties. Ponomar et al. [41] stated that, at 200 °C, Fe-rich alkali-activated materials undergo minor microstructural rearrangements, resulting in relatively stable mechanical performance with only slight strength variations associated with changes in porosity and initial matrix densification.

**Figure 10:**
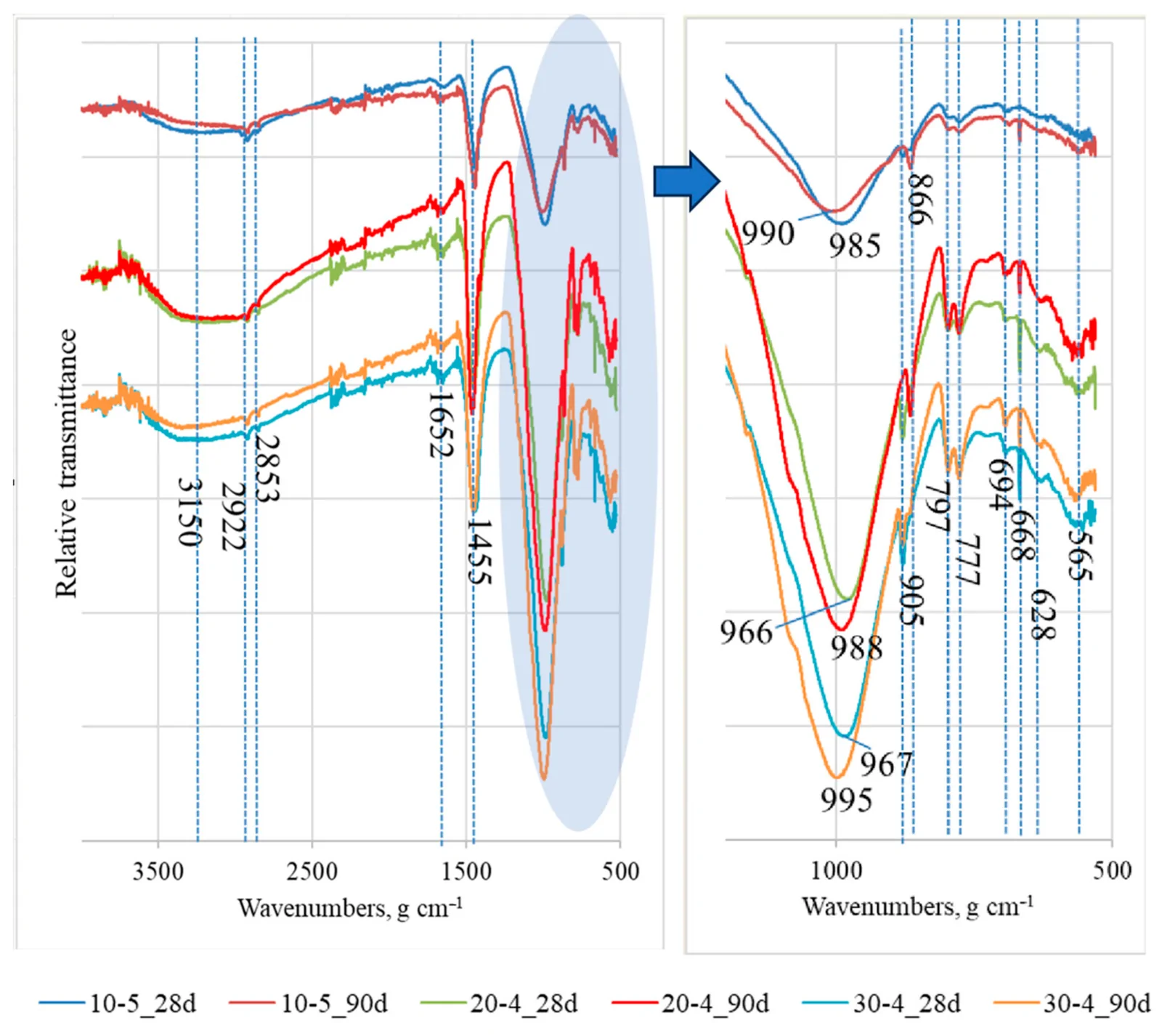
FT-IR spectra of alkali-activated binders (10-5, 20-4, and 30-4) with different amounts of BFA content before and after thermal exposure. The blue arrow points to a zoomed-in view of the blue area; the blue dashed lines are the vertical lines of the peaks.

Since the mineral composition identified by XRD analysis showed only minor differences, FT-IR analysis was performed on the same selected samples. All six samples exhibited similar FT-IR peaks; however, notable differences were observed in peak intensities (Figure 10).

The bands observed at approximately 3150 cm^−1^ can be attributed to the asymmetric stretching vibrations of the O–H group, while the bands near 1652 cm^−1^ correspond to the bending vibrations of the same functional group in absorbed water [42]. The intensity of both bands is significantly lower in the 10-5 samples with the lowest BFA inclusion (BFA:DT = 1:9) after 28 days of curing and following thermal exposure at 200 °C, compared with the samples containing higher amounts of BFA (BFA:DT = 2:8 and BFA:DT = 3:7). It is likely that BFA participates in the alkali-activation process and promotes the formation of gel phases such as N-A-S-H and C-A-S-H, which in turn increases the amount of hydroxyl groups incorporated into the structure, thereby contributing to the higher intensity of O–H-related FT-IR bands in samples with greater BFA content. Granizo et al. [43] stated that the addition of calcium resulted in higher aluminosilicate dissolution and a greater degree of geopolymerisation reactions.

All the analysed spectra exhibited peaks at wavenumbers of 1455 cm^−1^ and 905, 866 cm^−1^. These bands are associated with C-O bonds. This carbonate group currently has an asymmetric stretching vibration, and the carbonate group of calcium carbonate. The bands at 797 and 777 cm^−1^ (double peak) and 694 cm^−1^ could be the vibrations of Si–O chains in quartz. The presence of carbonates and quartz is likely related to unreacted phases originating from precursor materials, such as BFA and DT. It should be noted that increasing the BFA content in the system leads to a progressive increase in the intensity of the FT-IR bands characteristic of CaCO_3_ and SiO_2_. As previously reported in the literature [44], the occurrence of carbonate and quartz phases is commonly attributed to unreacted compounds remaining from the raw materials of geopolymers. Furthermore, weak bands appearing at 668 and 628 cm^−1^, together with a band near 565 cm^−1^, are indicative of bending vibrations associated with silicate and aluminosilicate frameworks, possibly overlapping with metal–oxygen vibrations from residual mineral phases [45].

The main absorption band observed at ~966–995 cm^−1^ is attributed to asymmetric stretching vibrations of Si–O–T bonds (T = Si, Al, Fe), indicating the formation of an aluminosilicate network in which iron may partially participate through incorporation into the geopolymeric gel structure. Similar bands at about 939–992 cm^−1^ were previously observed by Kaze et al. [46], who associated them with the formation of a Si–O–T (T = Si, Al, Fe) framework and the progressive development of the geopolymeric network. In all cases, after thermal exposure at 200 °C, the main bands shift toward higher wavenumbers, from 985 cm^−1^ to 990 cm^−1^, from 966 cm^−1^ to 988 cm^−1^, and from 967 cm^−1^ to 995 cm^−1^ for samples 10-5, 20-4, and 30-4, respectively. This shift is commonly associated with an increased degree of polymerisation and progressive structural reorganisation of the aluminosilicate network, reflecting the formation of a more condensed geopolymeric gel structure [47]. The intensity of both bands (10-5_28 and 10-5_90d) is significantly lower in the samples with the lowest BFA inclusion (BFA:DT = 1:9) after 28 days of curing and following thermal exposure at 200 °C, compared with the samples containing higher amounts of BFA (BFA:DT = 2:8 and BFA:DT = 3:7). It is likely that BFA participates in the alkali-activation process and promotes the formation of gel phases such as N-A/F-S-H and C-A/F-S-H, which in turn increases the amount of hydroxyl groups incorporated into the structure, thereby contributing to the higher intensity of O–H-related FT-IR bands in samples with greater BFA content. Furthermore, calcium present in BFA may enhance the dissolution of aluminosilicates and facilitate geopolymer gel formation, as reported by Canfield et al. [48]. The higher amount of geopolymeric gel results in the formation of a denser matrix and may promote the development of crystalline phases such as andradite, both of which contribute to enhanced mechanical strength. This is confirmed by the increased strength observed in samples 20-4_90d and 30-4_90d after thermal exposure at 200 °C, compared to their respective strength values before heat treatment. A different trend was observed for the 10-5 samples with the lowest BFA content. Thermal treatment led to a reduction in the intensity of the main band, accompanied by a decrease in compressive strength from 53 to 33 MPa. This may be attributed to partial recrystallisation of the geopolymeric gel into andradite, while the formation of microcracks could represent an additional factor contributing to strength loss.

### 3.3. SEM Microstructure of Alkali-Activated Binders as a Function of BMA Content

Figure 9 presents SEM micrographs of samples 10-5, 20-4, and 30-4 after thermal treatment at 200 °C, highlighting the relationship between microstructural features and compressive strength development.

**Figure 11:**
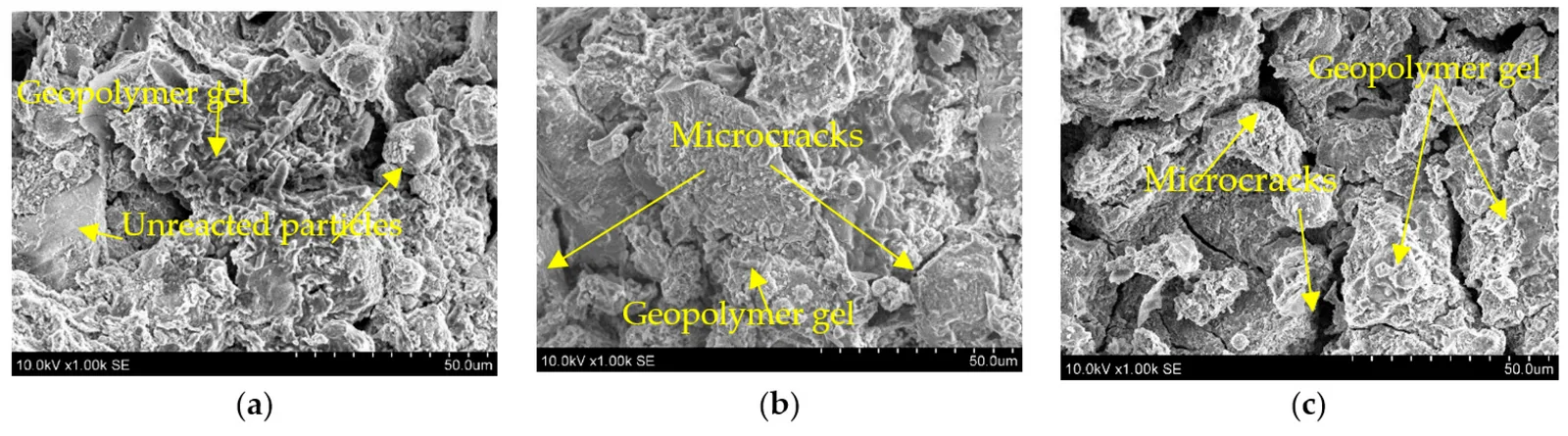
SEM micrographs of alkali-activated binders (**a**) 10-5, (**b**) 20-4, and (**c**) 30-4 containing different BFA contents after thermal exposure at 200 °C.

The microstructure of the 10-5_90d sample with the lowest amount of BFA (Figure 11a) is characterised by a relatively heterogeneous matrix, where geopolymer gel coexists with partially unreacted particles of the composite precursor. This limited degree of reaction results in weak interparticle bonding, which is reflected in a decrease in compressive strength from 53 MPa to 33 MPa after thermal exposure. The presence of local discontinuities further indicates reduced structural integrity. These observations are consistent with the XRD results, which show predominantly unreacted crystalline phases (quartz and magnetite), indicating limited participation of the precursor in the reaction process. Furthermore, FTIR analysis reveals lower intensity of the main Si–O–T band and hydroxyl-related bands in this sample, suggesting a lower degree of geopolymerisation and reduced gel formation. The combined XRD and FTIR findings therefore support the SEM observations and explain the inferior mechanical performance of the 10-5 system.

In contrast, sample 20-4_90d (Figure 11b) exhibits a denser and more homogeneous geopolymer gel matrix, indicating a higher degree of geopolymerisation. Although microcracks are present, their impact appears to be partially mitigated by the well-developed gel network, resulting in improved load transfer and more stable compressive strength compared to low-BFA systems. However, despite this refined microstructure, its compressive strength remains lower than that of sample 10-5_90d. This can be attributed to the higher BFA content, which reduces the proportion of reactive amorphous phases. XRD analysis confirms that a significant fraction of crystalline phases, such as quartz and magnetite, remains unreacted and acts primarily as inert fillers, limiting their contribution to strength development. Furthermore, FTIR results indicate increased intensity of carbonate- and O–H-related bands, suggesting the formation of Ca-rich phases and a less efficiently polymerised aluminosilicate network. Consequently, the combined effect of reduced geopolymerisation efficiency and microstructural defects results in lower compressive strength compared to the 10-5_90d sample.

For sample 30-4_90d (Figure 11c), the microstructure reveals an extensive network of microcracks within the geopolymeric gel matrix. Despite the formation of reaction products, the higher crack density acts as stress concentration sites, significantly compromising structural continuity and limiting compressive strength. This observation is consistent with the overall trend of decreasing strength at higher BFA contents. FTIR analysis indicates increased intensity of carbonate- and O–H-related bands, reflecting a higher content of Ca-rich phases, while the shift of the main Si–O–T band suggests ongoing polymerisation. However, due to the reduced availability of reactive amorphous Si at higher BFA content, the resulting geopolymeric network remains less effective in terms of mechanical performance.

Overall, the SEM observations confirm that compressive strength is strongly governed by the balance between gel formation and microstructural defects: a dense and continuous geopolymer gel enhances strength, whereas microcracking and unreacted particles reduce mechanical performance.

## 4. Conclusions

- The more homogeneous structure of the geopolymer gel is associated with higher mechanical properties, confirming that gel formation plays a key role in the strength development process. Compressive strength decreased from 53 to 33 MPa at 10% BFA after thermal treatment due to local discontinuities, while increasing the BFA content to 20% and 30% increased the softening factor and water resistance, but the compressive strength decreased to less than 20 MPa and 10 MPa, respectively, indicating a clear trade-off between strength and durability.
- XRD analysis revealed similar mineralogical compositions in all samples, dominated by largely unreacted quartz and magnetite, which act mainly as inert fillers, while thermal treatment at 200 °C promoted the formation of minor amounts of andradite without significantly altering the overall phase assemblage. FTIR results confirmed that increasing BFA content led to higher intensities of O–H and carbonate bands and a shift of the Si–O–T (T = Si, Al, Fe) band (~966–985 cm^−1^ to ~988–995 cm^−1^), indicating enhanced polymerisation; however, the coexistence of Ca-rich and partially unreacted phases resulted in a less optimal geopolymeric network.
- The samples with the lowest amount of BFA (10%) are characterised by a relatively heterogeneous matrix, where geopolymer gel coexists with partially unreacted particles of composite precursor. The sample with 20% BFA exhibits a denser and more homogeneous geopolymer gel matrix, indicating a higher degree of geopolymerisation. Although microcracks are present, their impact appears to be partially mitigated by the well-developed gel network, resulting in improved load transfer and more stable compressive strength compared to low BFA systems. The formation of Ca-rich phases and a less efficiently polymerised aluminosilicate network. Consequently, the combined effect of reduced geopolymerisation efficiency and microstructural defects results in lower compressive strength. For samples with 30% BFA, the microstructure reveals an extensive network of microcracks within the geopolymeric gel matrix. Despite the formation of reaction products, the higher crack density acts as stress concentration sites, significantly compromising structural continuity and limiting compressive strength. However, due to the reduced availability of reactive amorphous Si at higher BFA content, the resulting geopolymeric network remains less effective in terms of mechanical performance.
- Overall, the results demonstrate that the performance of alkali-activated binders is governed by the balance between gel formation, phase composition, and microstructural integrity, with optimal properties achieved at moderate BFA content rather than at the extremes.

## Figures and Tables

**Table 1:** Chemical composition of oxides in the raw materials based on XRF analysis, wt%.

	SiO_2_	CaO	K_2_O	Al_2_O_3_	MgO	SO_3_	P_2_O_5_	Fe_2_O_3_	Others	LOI
DT	85.05	-	1.84	7.74	1.44	-	-	3.18	0.74	-
BFA	32.99	32.63	7.48	5.91	5.57	5.38	4.26	2.87	2.91	0.42
ID	-	-	-	-	-	-	-	97.10	2.90	-

**Table 2:** Particle size characteristics of biomass fly ash (BFA), diatomite (DT) and iron dust (ID).

	BFA	DT	ID
d_10%,_ µm	1.34	6.33	2.01
D_50%,_ µm	20.27	35.51	21.41
D_90%,_ µm	167.62	84.41	107.55
mean diameter, µm	58.59	41.42	44.61
Specific surface area, cm^2^/g	3958	1959	3754

**Table 3:** Mix designs of alkali-activated materials.

Type	BFA Weight, %	ID Weight, %	DT Weight, %	Weight Ratio NaOH/(BFA + IS + DT)	NaOH Solution Concentration, mol/L	Weight Ratio Water/(BFA + IS + DT)
BFA:DT = 1:9						
10-1	4.4	56.1	39.5	0.09	5.7	0.41
10-2	5.2	47.9	46.9	0.08	4.5	0.44
10-3	5.9	41.5	52.7	0.07	3.6	0.48
10-4	4.4	56.1	39.5	0.14	8.5	0.41
10-5	5.2	47.9	46.9	0.12	6.8	0.44
10-6	5.9	41.5	52.7	0.11	5.7	0.46
10-7	4.4	56.1	39.5	0.19	11.5	0.40
10-8	5.2	47.9	46.9	0.16	9.7	0.41
10-9	5.9	41.5	52.7	0.14	7.2	0.49
BFA:DT = 2:8						
20-1	9.1	54.4	36.5	0.08	4.8	0.41
20-2	10.8	46.1	43.2	0.06	3.5	0.45
20-3	12.1	39.6	48.3	0.05	2.7	0.48
20-4	9.1	54.4	36.5	0.12	7.6	0.41
20-5	10.8	46.1	43.2	0.10	5.9	0.44
20-6	12.1	39.6	48.3	0.09	4.6	0.47
20-7	9.1	54.4	36.5	0.17	10.3	0.41
20-8	10.8	46.1	43.2	0.14	8.6	0.42
20-9	12.1	39.6	48.3	0.12	6.8	0.45
BFA:DT = 3:7						
30-1	14.2	52.5	33.2	0.07	4.0	0.41
30-2	16.8	44.1	39.1	0.05	2.7	0.44
30-3	18.7	37.5	43.7	0.03	1.6	0.51
30-4	14.2	52.5	33.2	0.11	6.7	0.41
30-5	16.8	44.1	39.1	0.09	5.0	0.43
30-6	18.7	37.5	43.7	0.7	3.8	0.44
30-7	14.2	52.5	33.2	0.15	10.5	0.36
30-8	16.8	44.1	39.1	0.12	7.3	0.42
30-9	18.7	37.5	43.7	0.10	5.8	0.43

## Data Availability

The original contributions presented in this study are included in the article. Further inquiries can be directed to the corresponding author.
